# Bacteriophages against *Salmonella enterica*: challenges and opportunities

**DOI:** 10.3389/fbioe.2025.1605263

**Published:** 2025-08-07

**Authors:** Nalleyt Segundo-Arizmendi, Alejandra Paola Flores-Coria, Janeth Gómez-Garcia, Efrén Hernández-Baltazar, Angélica Meneses-Acosta

**Affiliations:** ^1^ Laboratory 9: Microbiology and Parasitology, Faculty of Pharmacy, Autonomous University of the State of Morelos, Cuernavaca, Morelos, Mexico; ^2^ Laboratory 3: Research Lab, Latinamerican University, Cuernavaca, Morelos, México; ^3^ Laboratory 1: Pharmaceutical Formulation, Faculty of Pharmacy, Autonomous University of the State of Morelos, Cuernavaca, Morelos, Mexico; ^4^ Laboratory 7: Pharmaceutical Biotechnology, Faculty of Pharmacy, Autonomous University of the State of Morelos, Cuernavaca, Morelos, Mexico

**Keywords:** *Salmonella enterica*, bacteriophages, phage therapy, regulation of bacteriophages, bacteriophages products

## Abstract

*Salmonella spp.* is the most common pathogen transmitted to humans through contaminated water and food. Due to its ability to infect both animals and humans, as well as the spread of antibiotic-resistant strains, this pathogen has become a priority for food and pharmaceutical industries. Consequently, research and development of treatments to combat infections caused by *Salmonella spp.* are ongoing. One of the most promising strategies is the phage therapy (PT) which is based on the use of very specific viruses that infect this pathogenic bacterium without any action over the host and which use has shown effectiveness. Now a days, at least 41 companies worldwide market phage therapy products mainly for use in the food sector to reduce the transmission chain of *Salmonella spp.* to humans. However, the complex production processes required to ensure product quality, stability, safety, and efficacy, as well as the need for regulatory frameworks for phage therapy, present limitations to the global application of this strategy seems to be a limitation to promote its use all over the world as a pharmaceutical product. Thus, this work presents a literature review on state-of-the-art of PT, analysing the opportunities and challenges that are present to consider such a therapy as an emerging treatment for antibiotic resistance of *Salmonella enterica.*

## 1 Introduction

*Salmonella spp.* belongs to the family *Enterobacteriaceae*. This bacterial genus mainly comprises two species: *S. bongori* and *Salmonella enterica*. The first one is not important for human health, attacking mainly some cold-blood animals. The latter includes six subspecies, where subspecie enterica (I) is the one responsible for most infections. It comprises around 2,500 pathogenic serotypes that affect animals and humans, causing infections such as gastroenteritis, typhoid fever, localized infections, and even bacteremia (Deng et al., 2025).

Extrapolating globally, it is estimated that between 200 million and 1 billion people could be infected, which would result in an annual public health expenditure of approximately 3.3 billion dollars (U.S. Food and Drug Administration, 2023). In 2021, an estimated 29.6 million cases of typhoid fever were reported, considering a case fatality rate of 0.96%, where South Africa and Southeast Asia were the most affected regions. Non-typhoidal *Salmonella* diseases account for 25% of infections worldwide. In Europe and America, 1,170,000 cases are estimated annually, while the economic losses, though significant, have yet to be quantified for human health, livestock, or the poultry industries (World Health Organization, 2023).

The U.S. Centers for Disease Control and Prevention (CDC) estimates that despite widespread awareness of the bacterium and the quality controls required for food production, outbreaks caused by it continue to occur. In the United States, outbreaks have been linked to prepared cake mixes, sausages, lettuce, and other foods, even during the COVID-19 pandemic (Centers for Disease Control and Prevention, 2024). In Mexico, the number of illnesses caused by this bacterium has not decreased, with over 60,000 cases reported in the last 5 years (Secretary of Health, 2024). Additionally, considering the high resistance to antibiotics exhibited by these microorganisms, the World Health Organization (WHO) has identified the urgent need to find effective alternatives to mitigate the risks and infections caused by *Salmonella*. The WHO has also warned that by 2050, existing antibiotics will no longer be sufficient to treat infections in humans, potentially causing up to 10 million deaths per year (de Kraker et al., 2016). This alarming scenario underscores the importance of developing strategies to reduce the impact of this microorganism on animal and human health, as well as its role as an environmental pollutant.

Historically, infections caused by *Salmonella* have been treated with antibiotics such as ampicillin, tetracycline, cephalothin, streptomycin, kanamycin, chloramphenicol, and trimethoprim with sulfamethoxazole. However, improper use in both human and veterinary medicine has facilitated the bacterium’s adaptation. As a result, multidrug-resistant *Salmonella spp*. has been reported in Europe, Asia, and America, often isolated from samples such as poultry and agricultural workers (V T Nair et al., 2018). Considering this huge worldwide problem, it is necessary to look for new strategies to combat antibiotic resistance. One of the most promising strategies to combat this issue is the use of bacteriophages, viruses that infect and kill bacteria specifically. This approach seeks to eliminate both infectious bacteria and contaminants in consumable products. Therefore, the present work aims to review the progress achieved in phage therapy against *Salmonella spp*.

## 2 Characteristics of *Salmonella spp.*

*Salmonella spp.* is gram-negative, motile, facultatively anaerobic, catalase-positive, oxidase-negative, non-sporulating, and non-encapsulated bacilli. These bacteria can ferment sugars and produce gas. Unlike other genera of the same family, *Salmonella spp.* can grows in the presence of brilliant green dye, sodium tetrathionate, and sodium deoxycholate, and they can withstand freezing and prolonged periods in water (Rahman et al., 2018; Oludairo et al., 2022).

This bacterial genus mainly comprises two species: *S. bongori* and *S. enterica*. This latter includes six subspecies: enterica (I), salamae (II), arizonae (IIIa), diarizonae (IIIb), houtenae (IV), and indica (VI). According to their clinical characteristics, the bacteria of this genus can be classified as *typhoidal Salmonella* (TS), *non-typhoidal Salmonella* (NTS), and *invasive non-typhoidal Salmonella* (iNTS) (Gordon, 2011; Gibani et al., 2018; Wang et al., 2020). The first group comprises *S. Typhi*, *S. Paratyphi*, and *S. Sendai*, which are the causative agents of typhoid fever, a disease characterized by fever up to 41°C, headache, drowsiness, and pea-like diarrhoea. The second group includes *S. Enteritidis, S. Dublin*, and *S. Typhimurium*, which can cause a range of infections from self-limiting gastroenteritis to severe conditions such as bacteremia and meningitis. Finally, the third group includes hundreds of serotypes; although recent outbreaks in Africa have been caused by *S. Enteritidis* and *S. Typhimurium*; invasive infections of this group are often associated with preexisting diseases such as malaria and can result in high fevers, respiratory distress and even death (Gordon, 2011; Gibani et al., 2018; Wang et al., 2020; Khan and Shamim, 2022).

## 3 Mechanism of *Salmonella spp*. infection

*Salmonella spp.* enters the host through the ingestion of contaminated food. The number of bacteria required to initiate an infection depends on the strain’s identity and the host’s susceptibility. However, it typically ranges from 10 to 100,000 colony-forming units (CFU) (Alves da Cunha Valini et al., 2023; Lamichhane et al., 2024). Once in the stomach, the bacteria can resist acidic pH due to proteins encoded by the *hdeA, hdeB, rpoS*, and *gadC* genes, as well as porins such as OmpA and OmpC, which regulate ion influx and contribute to bacterial survival. Additional survival mechanisms include proton pumps, the enzyme glutamate decarboxylase, and vacuoles formed via the Type III secretion system, all of which enhance the bacteria’s ability to persist in this hostile environment (Lamichhane et al., 2024).

Once the bacteria reach the small intestine, they internalize into M cells and enterocytes with the help of proteins encoded on the *Salmonella* Pathogenicity Island I (SPI-1). Using the Type III Secretion System (SSTT), often referred to as “*molecular needle*”, bacteria deliver effector proteins, including SipA, SipC InvA, and SopE. These proteins reorganize the host cell’s cytoskeleton, promoting the curling of intestinal microvilli (ruffling), which facilitates bacterial entry into the cell. Once inside, *Salmonella* is able to infect adjacent cells on both the apical and basolateral sides, likely through a similar cytoskeleton rearrangement (Lhocine et al., 2015). In the typhoidal varieties, *Salmonella* can internalize into macrophages located in Peyer’s patches. Within these macrophages, bacteria use a second SSTT, encoded on *Salmonella* Pathogenicity Island 2 (SP2), to inhibit the phagolysosome function. For instance, the SifA and SifB proteins produce a filament that helps *Salmonella* attach itself to the membrane and evade lysosome action. Additionally, PipB facilitates cytoskeleton rearrangement, enabling Sif activity, while SseA and SseB further prevent the binding of *Salmonella* to the phagolysosome (Lamichhane et al., 2024).

Additionally, some proteins encoded on virulence plasmids also promote the survival of the bacteria. For example, SpvB and Spv interrupt macrophage apoptosis, allowing bacteria to survive and be transported within these phagocytic cells. As a result, *Salmonella* does not enter to the phagolysosome but instead remains within the macrophage. This change in environment promotes the replacement of fimbriae, which enables the bacteria to evade the immune system and facilitates biofilm formation. It is interesting to highlight that the involved genes are located on the bacterial chromosome (Lamichhane et al., 2024).

During invasion, the host attempts to stop the infection by releasing pro-inflammatory cytokines such as IL-1, IL-6, and Tumor Necrosis Factor-alpha (NTF-ɑ), which causes vasodilation. As a result, a many immunological cells are concentrated at the site of infection, helping to destroy bacteria. However, this response also contributes to intestinal damage by increasing permeability. Once the infection is controlled, anti-inflammatory interleukins appear to help prevent excessive inflammation (Gut et al., 2018; Lamichhane et al., 2024).

## 4 *Salmonella* resistance to antibiotics

Historically, ampicillin, trimethoprim, and sulfamethoxazole were first-choice treatments for ST infections (Crump et al., 2015). In the case of infections caused by NTS, these are generally self-limited; however, when antibiotics are necessary, options such as ciprofloxacin, trimethoprim with sulfamethoxazole, and ampicillin have been employed (Crump et al., 2015). Unfortunately, bacterial resistance to antibiotics has complicated the treatment of such infections. Over the last decade, bacteria resistant to tetracyclines, nadixilic acid, and ampicillin have been found not only in patients but also in bacteria isolated from food products such as red meat, poultry, and even eggs. Therefore, several researchers have sought alternatives for treating and preventing these diseases, one of which is the use of Bacteriophages (Khan and Shamim, 2022).

Recent studies have warned about the high bacterial resistance to antibiotics presented by *Salmonella* strains. For example, Fatima and cols. conducted a 2021 survey of *Salmonella enterica* in raw meat in Pakistan. They collected 111 samples, of which a genus of this bacterium was identified in 57 (51.35%) of them, all presenting resistance to antibiotics. The most common resistances were to cephalosporins, macrolides, sulphonamides, and aminoglycosides (Fatima et al., 2023). These findings align with other studies, which suggest that the transmission of IncFII, Incl1, Incl2, and InclB/O/K/Z plasmids is the cause of the dissemination of this resistance (Yang C. et al., 2023).

For a long time, the first-choice treatment against *Salmonella* was trimethoprim with sulfamethoxazole. However, since 1980, this bacterium has developed resistance to this antibiotic. Additionally, resistance has also been observed to antibiotics from the following families: Amphenicols (Chloramphenicol), Aminoglycosides (Kanamycin, Streptomycin), Carbapenems (Meropenem), Cephalosporins (cefepime), Fluoroquinolones (Ciprofloxacin), Macrolides (Erythromycin and Azithromycin), Penicillins (Ampicillin), Polymyxin (Colistin), Polypeptides (Polymyxin), Quinolones (Nalidixic Acid), Sulphonamides (Trimethoprim), Tetracycline (Tetracyclone, Doxycycline) (Yang C. et al., 2023; Lamichhane et al., 2024).

Although agriculture and environmental conditions have influenced the distribution of *Salmonella*, certain varieties have distinct geographic ranges. For example, *S. Heidelberg* and *S. Newport* are only found in North America, while the variety *S. Agona* has been reported exclusively in Europe. In contrast, *S. Typhimurium* and *S. Enteritidis* are globally distributed. These two varieties show significant antibiotic resistance, with some strains classified as multidrug-resistant, meaning they fail to respond even to the most effective antibiotics currently available. For this reason, the World Health Organization (WHO) has classified *Salmonella* strains resistant to cephalosporins as a priority on its list of bacteria requiring new therapeutic options. This classification considers the ubiquity of the bacteria, the incidence, prevalence, severity, mortality, transmissibility, and treatment challenges associated with the infections they cause (Lamichhane et al., 2024; World Health Organization, 2024).

## 5 Bacteriophages

Bacteriophages, also known as phages, are viruses that specifically infect bacterial strains of a given species. They are found in many environments and are considered the most abundant entities on Earth, with an estimated total of 1 × 1031 plaque forming units per millilitre (PFU/mL). Bacteriophages are composed of genetic material (DNA or RNA) and proteins, and they exhibit remarkable diversity in size, morphology, and genetic organization. Their structures may include or not a capsid, neck, tail or cauda, basal lamina, and/or spicules (Sulakvelidze et al., 2001; Zhu et al., 2022). While most phages have a tail, some possess membranous envelopes from their host. This diversity led the International Committee on Viral Taxonomy (ICVT) to recognize at least 19 families of such viruses that infect bacteria in 2022.

It is well known that bacteriophages can also be categorized based on their replication cycle, which always begins with the recognition of bacterial receptors and the introduction of the viral genetic material into the bacterial cell. On the one hand, bacteriophages that follow the lytic cycle immediately start replicating their genetic material and synthesizing enzymes that facilitate this process (sometimes including their polymerase and structural proteins). This process leads to the assembly of the virus and its subsequent release from the host cell. On the other hand, bacteriophages undergoing the lysogenic cycle insert their genetic material into the bacterial chromosome, where it behaves as another gene instead of immediately initiating their replication. Furthermore, if the lysogenic bacterium replicates, its progeny cells will inherit the viral genetic material (prophage) within their genome. This state can persist for many generations until the bacterium undergoes a stressful process, called the induction process, which triggers the viral genome to exit the chromosome and replicate, resuming the lytic pathway (Sulakvelidze et al., 2001; Ling et al., 2022; Zhang et al., 2022).

These characteristics prioritize lytic bacteriophages as a potential alternative for treating bacterial infectious diseases through phage therapy (Gordillo Altamirano and Barr, 2019). Bacteriophages have been used to eliminate different bacteria in contexts including aquariums, food, machinery, operating rooms, and even in animals and humans via topical, enteric, and systemic routes. This is because they offer two key advantages over antibiotics: first, their high specificity allows them to target only the causative agent of the infection, eliminating the risk of dysbiosis and adverse effects, making them a safe therapeutic option; second, their viral replication cycle guarantees that a single phage dose can result in continuous viral production as long as susceptible bacteria are present, generating a self-sustaining process that allows treatments of shorter duration compared to antibiotics. Once the susceptible bacteria are eliminated, the immune system clears the bacteriophages from the organism (Kaliniene et al., 2017; Luong et al., 2020).

## 6 History of bacteriophages

Bacteriophages were identified by Ernest Hankin in 1896 when he observed small zones of no growth of *Vibrio cholerae* in bacterial cultures containing water from India’s Ganges and Jumna rivers. Hankin suggested that an undefined substance caused the pathogen’s growth inhibition (Golkar et al., 2014). Other contemporary researchers described similar phenomena, which they termed “*bacterial autolysis*”. However, their descriptions, not being in English, limited their visibility. This was the case for Gildemeister, Summers, Emmerich, and Low, until Frederick Twort revisited the subject in 1915 (Abedon et al., 2011). In 1917, Félix d'Hérelle, a French-Canadian scientist, introduced the term bacteriophages to refer to a potential parasite found in faecal filtrates from dysentery-afflicted soldiers (Summers, 2016) This agent could lyse the causative bacterium and reportedly cured patients after a single administration (Dublanchet and Bourne, 2007).

Following this discovery, between 1920 and 1940, numerous researchers-initiated studies on phage therapy. The Tbilisi Institute in Georgia was founded in 1923, and it continues operating today as one of the world's most significant centers for phage therapy, capable of diagnosing and treating various bacterial infections in a species-specific manner. In the Western hemisphere, Brazil was a leading country in phage therapy research. Dr. Oswaldo Cruz established an institute that, from its inception, also investigated and treated patients using phage therapy. The Oswaldo Cruz Foundation continues to research alternative treatments for infectious diseases, biological and biotechnological products, epidemiological surveillance, and other public health areas (Almeida and Sundberg, 2020). However, in these countries, bacteriophages fell into disuse due to an incomplete understanding of their nature, the publication of contradictory treatment results, and the emergence of antibiotics. Another example illustrating the decline of phage therapy after the 1940s is the company L’Oréal in France, which previously sold at least five bacteriophage-based products. This company subsequently shifted its focus to the cosmetics industry (Sulakvelidze et al., 2001). By the 1960s, the replication strategies, structure, and composition of viruses were elucidated. Since then, advancements in phage therapy have broadened to include various bacterial targets and diverse delivery strategies, with applications across agricultural, livestock, veterinary, and human medicine industries.

## 7 *Salmonella* eradication using bacteriophages

Currently, there is no record of the number of isolated bacteriophages worldwide capable of lysing *Salmonella enterica* However, virtual platforms such as National Library of Medicine and Science Direct report 42,986 and 15,364, resulting from the same search respectively. These databases also show a growing number of articles published on these topics, reaching more than two hundred publications per year. Researchers in the East have increased their publications regarding phage therapy, particularly in relation to treating *Salmonella* spp. in animals, humans and agriculture, driven by the bacteria’s ubiquity and its ability to infect different hosts.

The strains against which bacteriophages have been found are diverse, being the most common: S. Paratyphi, S. Ttyphi, S. Typhimuriun, S. Gallinarum, S. Cholerasuis, S. Enteritidis and S. Pullorum. The names of some of the bacteriophages isolated and characterized against this genus in the last 5 years are listed in Supplementary Table S1. It is noteworthy that bacteriophage characterization studies typically focus on biological components (plaque morphology, microscopic morphology, viral structure, growth curve, host breadth, propagation characteristics) and genetic components (host identity determination, sequence analysis, lysogeny genes search, gene identification).

## 8 Phage therapy against *Salmonella spp.* in industries

Table 1 summarizes the bacteriophage-based products being investigated for use in PT (Bacteriophage.news, 2024). However, many other products are being studied (Fiorentin et al., 2005; Hungaro et al., 2013; Huang et al., 2018; Shang et al., 2021; Mhone et al., 2022; Yousefi et al., 2023). The administration strategies of bacteriophages against *Salmonella* vary, with liquid preparations being the most common presentation (Fiorentin et al., 2005; Hungaro et al., 2013). Other forms include lyophilized and encapsulated bacteriophages, which are suitable for oral administration or, in the case of food, for spraying on meat or agricultural products. Despite the importance of this bacterium in pets, there are no reports of *Salmonella*-based products used as antiseptics for cleaning of fish tanks or areas where pets are kept (Bacteriophage.news, 2024).

**TABLE 1:** Bacteriophage-based products against *Salmonella spp.* are being marketed and researched worldwide.

Product	Target strain	Formulation	Title	Company/Link	Country
GastroPHAG	*Salmonella* Typhimurium *Salmonella* Newport *Salmonella* Enteritidis *Salmonella* Moscow *Salmonella* Paratyphi B *Salmonella* Agama *Salmonella* Java	Lyophilized bacteriophages stored in soft gelatine capsules	1X104	Aziya inmunopreparat LLC https://aziyaimmunopreparat.uz	Uzbekistan
Fhagesti	Shigella spp *Salmonella* spp *Escherichia coli* *Proteus* spp *Enterococcus* spp *Pseudomonas* spp	Liquid	ND	Biochimpharm https://biochimpharm.ge/en/product/phagesti-4x-20ml/	Georgia
Septaphage	*Shigella* spp *Salmonella* spp *Escherichia coli* *Proteus* spp *Enterococcus* spp *Staphylococcus* spp *Pseudomonas* spp	Tablet	ND	Biochimpharm https://biochimpharm.ge/en/products/	
TravelphageTM	*Shigella* spp. *Salmonella* spp. *Escherichia coli* *Proteus* spp *Staphylococcus* spp *Pseudomonas* spp. *Enterococcus* spp.	Capsule	ND	Biochimpharm https://biochimpharm.ge/en/products/	
Intesti Bacteriophage	*Salmonella* Paratyphi A *Salmonella* Paratyphi B *Salmonella* Enteritidis *Salmonella* Cholerasuis *Salmonella* Oranienburg	Liquid	ND	Eliava https://phage.ge/en/products	Georgia
ENKO Bacteriophage	*Salmonella* spp.	Liquid	1.X105	Eliava https://phage.ge/en/products/enko-bacteriophage	
In development	*Salmonella* spp.	ND	ND	Jafral https://jafral.com/bacteriophages-phages/	Slovenia
Phage preparations and dressings	*Salmonella* spp.	ND	ND	Hirszfeld Institute https://hirszfeld.pl/en/structure/iitd-pan-medical-center/phage-therapy-unit/phage-therapy-basis/	Poland
Intesti Bacteriophage	Salmonella Paratyphi A *Salmonella* Paratyphi B *Salmonella* Typhimurium *Salmonella* Enfants *Salmonella* Cholerasuis *Salmonella* Oranienburg *Salmonella* Enteritidis	Liquid	ND	Microgen /https://www.microgen.ru/en/products/bakteriofagi/intesti-bakteriofag/	Russia
Phageguard S	*Salmonella* spp.	Liquid	ND	Microos Food Safety BV (Phageguard) https://phageguard.com/solutions/salmonella?gad_source=1&gclid=EAIaIQobChMI04CH8PWshwMVxJiDBx1llgs3EAAYASAAEgLBm_D_BwE	Netherlands
Phageguard S5c	*Salmonella* spp.	Liquid	ND		
Intestifag	*Salmonella* enterica	Liquid	ND	Phagex /https://bacteriophages.info/en/bacteriophage/intesifag/	Ukraine
INSPEKTOR®	*Salmonella* spp	Liquid	ND	Phage-Lab ( PhageLab - Science works and we can prove it)	Brazil
Phage in	*Salmonella* spp and *Escherichia coli*	Powder additive	ND		

The poultry industry has the highest number of bacteriophage formulations on the market, likely due to the limitations in European regulations on the use of antibiotics as a necessary measure to prevent the spread of antibiotic-resistant bacteria, or perhaps because bacteriophages can be easily mixed with poultry feed (Suresh et al., 2018; Chen et al., 2019).

The use of bacteriophages in poultry has been a recurring theme in the scientific literature. Notably, the results reported by Hungaro et al. (2013) and Fiorentin et al. (2005), despite employing different methodological approaches, both demonstrated significant reductions of *Salmonella spp*. strains in ready-to-eat poultry meat trials. Similarly, Gómez-García et al. (2021) showed that an alginate-encapsulated phage managed to eliminate the bacteria in live birds. Further advancing this field, Aguilera et al. (2023) independently achieved reductions of up to six logarithmic units in *Salmonella* contamination across various serovars using the bacteriophage cocktail known as INSPEKTOR^®^ in Brazil, successfully treating three million chickens (Pino et al., 2025).

Currently, there are ten products available against *Salmonella* for use in farm chickens, two for cattle and at least one for pigs and agricultural use. As part of efforts to prevent and control of *Salmonella*, the company Biochimpharm has developed two bacteriophage concentrates: PHAGESTI™ y SEPTAPHAGE™ for pharmaceutical use in humans, with more information available on their website: https://biochimpharm.ge/en/products/. In the swine and vaccine industries, the implementation of bacteriophage cocktails has begun, showing significant reductions in S. Enteritidis infections (Sriprasong et al., 2022; Thanki et al., 2022; Nale et al., 2023).

One of the major challenges presented at the technological level is the production and formulation of bacteriophages. Bacteriophages used in phage therapy undergo purification processes that typically include deep filtration, chromatography, dialysis filtration, sterility controls, endotoxin testing and identity and potency tests, in addition to sequencing. Due to their physicochemical characteristics, bacteriophages are sensitive to various processes, making liquid presentations the easiest pharmaceutical form to be produced and the most common in industry. However, this complicates the product stability and its shelf life. Furthermore, when considering that bacteriophages in oral presentations against *Salmonella* must not only remain infectious against the bacteria but also pass through the host’s gastrointestinal tract to reach the intestinal area where the bacteria reside, the challenge becomes more apparent. To address this, several authors have used freeze-drying, spray drying and encapsulation as primary strategies to create solid products, such as gels, emulsions, microcapsules, sprays, and suppositories (Straka et al., 2022). Table 2 shows the excipients used in bacteriophage formulations, classified by way of administration, that have been employed by different authors.

**TABLE 2:** Excipients used in bacteriophages by route of administration.

Administration route	Formulation	Excipient	References
Topic	Gels	Hydroxypropyl methylcellulose (HPMC) Hydroxymetylcellulose (HMC) Gelatin Sodium Alginate Glycerin Sorbitol Benzoic acid and parabens	Abdelsattar et al. (2019) Mabrouk et al. (2022) Moghtader et al. (2024) Gónzalez-Menendez et al. (2018) Narayanan et al. (2024)
	Emulsions	Polysorbates Glyceryl stearate Tween 80 Span 20 Polyethylene glycol Glycerin and sorbitol Benzoic acid and parabens	Rastogi et al. (2017) Narayanan et al. (2024)
Oral	Microcapsules	Alginate Chitosan Gelatin Synthetic polymers (Polyethylene glycol and Polylactic acid) Maltodextrin and sorbitol	Gómez-García et al. (2021) Malik et al. (2017) Hu et al. (2024) Muhoza et al. (2020) Yongsheng et al. (2008)
Respiratory tract	Spray	Salt Potassium Chloride Calcium Magnesium Fluoride Sulphate Calcium carbonate Nitrate	Wessels et al. (2022)

Thus, it was possible to make alginate microspheres containing the bacteriophage Felix 01, achieving an encapsulation rate of 93.3% and an infectivity rate of 12.6%, after 6 months of storage at 4°C (Yongsheng et al., 2008). Also, Lorenzo-Rebenaque et al. (2022) demonstrated that by encapsulating the bacteriophage Φ and adding Eudragit L100 it was possible to eliminate contamination in a poultry farm within just one production cycle.

## 9 Regulatory framework of phage therapy

PT has been used since the discovery of bacteriophages in Eastern Europe and the former Soviet Union, primarily as a treatment for gastrointestinal and cutaneous bacterial conditions (Sulakvelidze et al., 2001; Furfaro et al., 2018). Despite current reports on PT, its application in human clinical therapy has been limited due to insufficient data on the safety and toxicity of bacteriophages, as well as the lack of regulation in this respect (Brives and Pourraz, 2020) to clearly establish identity, safety and efficacy criteria for products ensuring their quality. However, it is important to highlight that the use of bacteriophages in agricultural, animal, and human treatments has been gradually increasing. International agencies have recognized bacteriophages as drugs, biologics, and biotechnological products (Brives and Pourraz, 2020), enabling the use of PT in healthy volunteers and patients infected with multidrug-resistant bacteria (MDR). Several countries have explored ways to introduce PT in compliance with current regulations. In the European Union (EU), bacteriophages have been classified as “medicinal products” and are regulated by the European Medicines Agency (EMA) under Directive 2001/83/EC (Faltus, 2024). Under this regulation, bacteriophages can be marketed as “industrial preparations”, for which it is necessary to develop production and quality standards to obtain EMA authorization. This has led to the development of specific regulation for bacteriophage-based products (Pirnay et al., 2018).

In Germany, bacteriophages are considered biological medicinal products under Directive 2001/83/EC, meaning they require market access authorization. The only exception to this is when bacteriophages are administered as a masterly formula for a specific patient. The use of bacteriophages in patients has been notably successful in Belgium. In fact, the literature often refers to “the Belgian model” as a framework to enhance the success of phage therapy, which improves the chances of success in phage therapy. This model consists of: i) acquiring bacteriophages from different institutions that have already isolated, investigated, purified and tested the effectiveness of the virus, and ii) using bacteriophages as a last-resort treatment for patients, all through a multidisciplinary approach (Willy et al., 2023). The same model is also followed in Poland at the Hirszfeld Institute of Immunology and Experimental Therapy, where specific bacteriophages are identified for each patient.

In France, the Agence Nationale de Sécurité du Médicament et des Produits de Santé allows PT under the “*compassionate use*” argument. This allows products that have not yet received market authorization in that country to be used as treatment option when antibiotic therapy fails in patients infected with MDR bacteria (Pirnay et al., 2018). In this field, Poland has made the greatest progress in establishing the PT as a standard treatment, incorporating it into the law on medical and dental professions enacted since 1996. Additionally, PT is included in the ethical code of the Polish Association, and as a member of the European Union, Poland also adheres to the EU directives 2001/83/EC and 2005/28/EC, which regulate good practices in clinical trials (Yang Q. et al., 2023).

In Georgia, the PT field has been advanced by the Eliava Institute of Bacteriophages, Microbiology and Virology, which was founded by D´Herelle. This institute commercialize phage preparations, making them available without prescription. As a result, phages are considered pharmaceuticals in Georgia, and PT is recognized as a standard medical treatment. In Australia, Phage Australia serves as a national alliance that organizes and standardizes the use of phage therapy (Yang Q. et al., 2023).

In China, bacteriophages have been used to treat infections since 1958. In 2019, the Phagos Institute in Shanghai conducted the first clinical trial with ethics committee approval. According to Chinese regulations, commercial applications of PT can follow two pathways: 1) phage products with fixed ingredients, which are regulated as bio-innovative products; and 2) customized products, which function as a kind of “*library*” from which specific phages can be ordered based on the patient’s needs (Johri et al., 2023; Yang Q. et al., 2023).

In the United States of America (USA), patients may receive PT following the guidelines for new drugs under emergency investigation by the Food and Drug Administration (FDA). This allows patients to access therapies involving unapproved drugs or biologics, if they meet the criteria for compassionate use of bacteriophages, as outline in the Article 37 of the Helsinki Declaration. Specifically, this applies if the infection poses an immediate life-threatening risk, there are no comparable medical alternatives, the potential benefits of the therapy outweigh the risks, and the use of bacteriophages does not interfere with ongoing clinical investigations (Hitchcock et al., 2023). In 2021, the FDA held a series of expert conferences to discuss the perspectives and regulations of PT. Such conferences emphasized the importance of promoting PT among researchers, doctors and decision-makers to promote legislation on bacteriophages. The FDA also recommended using product frameworks such as vaccines, viral vectors, neutralizing antibodies, and cellular immunotherapy as models for developing regulations in this pharmaceutical sector (Yang Q. et al., 2023).

**FIGURE 1:**
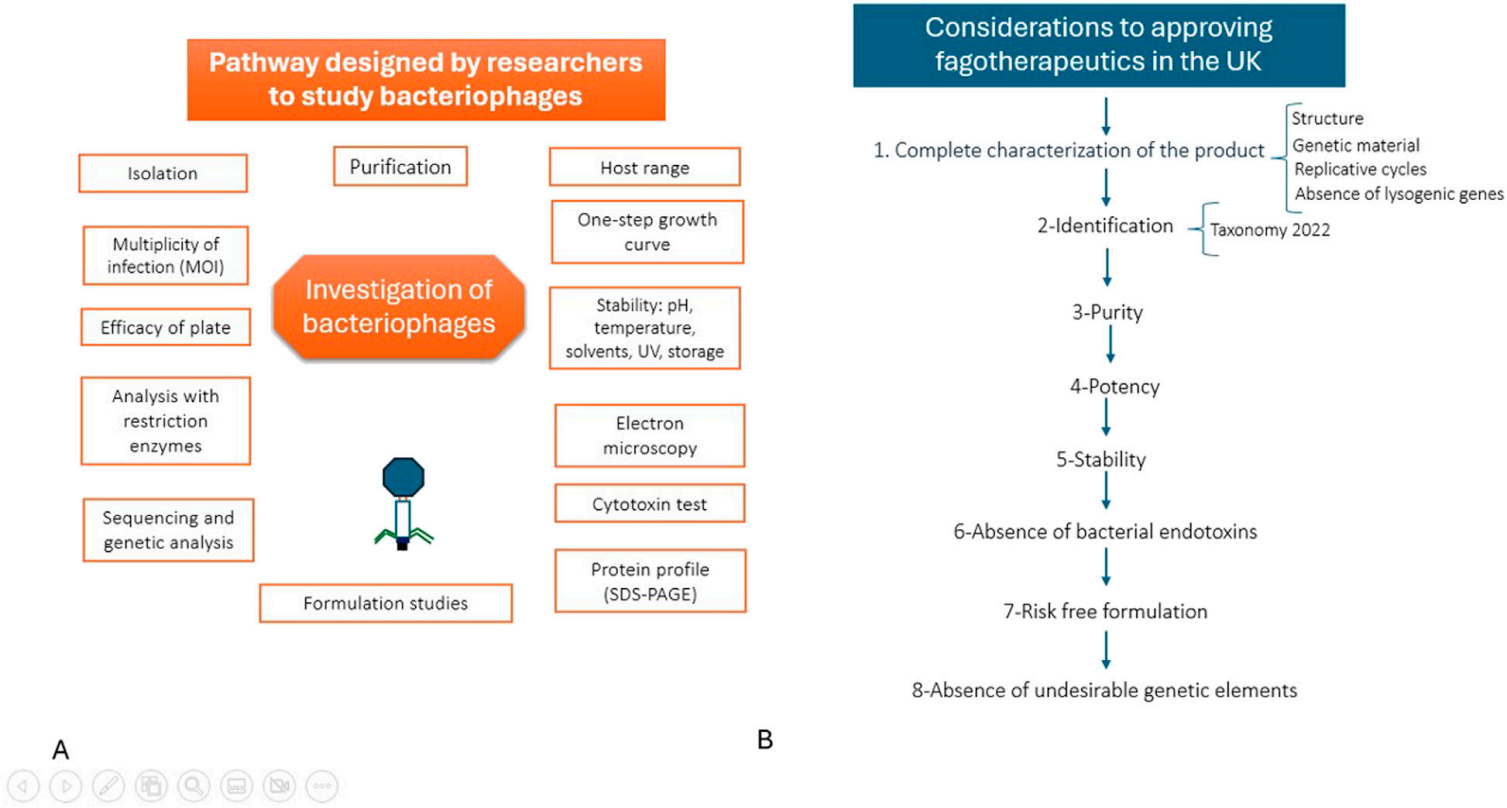
Development and regulatory evolution of bacteriophage therapy. A brief description of the discovery process for bacteriophages developed over the years in different countries **(A)**, as well as novel information regarding regulation in phage therapy **(B)**, is presented.

Considering the importance of bacterial resistance to antibiotics, the regulation of fags’ use by governmental authorities is a becoming urgent necessity and treatment alternatives must be prepared and standardized before the emergency escalates and it seems that PT is starting to get some attention for governments and regulatory agencies again. Recently, on 4 June 2025, the United Kingdom issued “*Regulatory considerations for therapeutic use of bacteriophages in the UK*,” a document that defines “bacteriophage medicinal product” as monovalent or polyvalent biological or biotechnological products. Their therapeutic activity stems from the fags’ ability to infect and lyse specific pathogenic bacteria. These products are administered to treat infections resistant to conventional antibiotics or in cases where conventional therapy has been ineffective. In this document, the authorizing conditions for a bacteriophage-based product for sale are indicated, which include: i) compliance with “Good Manufacturing Practices (GMP),” ii) complete characterization of the product, iii) identification, iv) purity, v) potency, vi) stability, vii) ensuring absence of bacterial endotoxins, viii) ensuring a risk-free formulation, ix) absence of undesirable genetic elements, all of which must be extensively documented. Although no product is currently licensed in the UK, the appearance of this regulatory guidance marks a significant step for the regulation, distribution, and dispensing of fagotherapeutics (Medicines and Healthcare products Regulatory Agency, 2025) and it is opening a new door for PT as a regulated, real and accessible alternative for bacterial resistance. It has to be noted that all the specifications that such regulatory guide includes have been previously stablished by researchers that develop the experimental work in this issue. In Figure 1, a brief description of the discovery process for bacteriophages developed over the years in different countries (Figure 1A), as well as novel information regarding regulation in phage therapy (Figure 1B), is presented. Figure 1 illustrates that research serves as the foundation for supporting new regulatory frameworks aimed at enabling the development of feasible commercial products.

In addition, the social acceptation of PT should be spread between scientific community and general population. For that, some efforts were done in 2015 and 2019, where an international phage course was held in Latin America, involving 84 students from 14 countries. The course focused on techniques for isolation, characterization, genomic sequencing and registration of new phages (Payaslian et al., 2021). The aim was to disseminate knowledge about PT in this geographical area. However, this does not mean that phages and PT are unknown in Latin America, as numerous *in vitro* and *in vivo* isolations and studies, mainly in animals, have reinforced the effectiveness of phages as reported in other countries. Nevertheless, more efforts are needed to help countries address the growing threat of infections by multidrug-resistant or pandrogoresistent bacteria, as warned by the World Health Organization (Aslam et al., 2021).

An increasing number of countries recognize the need to develop policies that guide the implementation of PT. This requires a deeper understanding of bacteriophage biology to address and combat bacterial resistance observed in certain bacteria. Additionally, it calls for conclusive clinical trials conducted under standardized guidelines, ethical practices and techniques to demonstrate the safety and efficacy of bacteriophages.

## 10 Clinical trials on phage therapy

Between 1992 and 2022, an estimated 6,300 patients were treated with PT in hospital departments such as pneumology, urology, orthopaedics, dermatology, otorhinolaryngology, gastroenterology, cardiology and palliative care. Bacteriophages have been used as a therapeutic alternative for bacterial genera including *Enterobacter* spp.*, Acinetobacter* sp.*, Pseudomonas* sp.*, Staphylococcus* spp.*, Salmonella spp., Shigella spp., Mycobacteria* spp.*, Burkholderia* spp.*, Serratia spp.* and *Neisseria spp*. Furthermore, phage therapy has been carried out on multiple continents, including Europe, America, Asia, and Africa (Diallo and Dublanchet, 2023).

Several countries have conducted clinical trials including PT. In 2017, Germany reported a trial with 170 participants from 20 countries, which helped promote open discussions on the topic. In 2019, the same country published a study with 67 participants from 17 nations, which supported the idea of establishing an international registry of bacteriophages to improve access to specific phages targeting bacteria. Similarly, in 2022, a study involving 100 participants from 8 countries led to the authorization of PT for use in hospitals (Willy et al., 2023).

In 2023, there were 25 clinical trials related to phage therapy, and in 2024, this number increased to 41. This growth reflects the rising interest in PT, driven by the threat of bacterial resistance to antibiotics, the effectiveness of bacteriophages in research, and their relatively few adverse effects compared to antibiotics (Hitchcock et al., 2023; Willy et al., 2023; Yang Q. et al., 2023). Table 3 describes the clinical trials related to bacteriophages in humans the most studied bacteria were *Pseudomonas aeruginosa* and *Escherichia coli*, with six studies respectively. The dosage used depends on each study, however, they range between 10^3^–10^9^ CFU/mL. The most common routes of administration were oral and inhalation. For *Salmonella spp.* the trials have only included animals such as poultry and pigs.

**TABLE 3:** Research and clinical trials of phage therapy in humans.

Country	Year	Development phase	Target bacteria	“n”	Formulation	Dosage	Route of administration	References
United States of America	2025	Phase I	*Escherichia coli*	N.E.	P.C.	N.E.	Intravesical	ClinicalTrials.gov, (2025a)
	2024	Phase II	*Pseudomonas aeruginosa*	48	P.C.	N.E.	Inhalable	ClinicalTrials.gov, (2024)
	2021	Phase II	*Pseudomonas aeruginosa*	8	M.S.	individualized	Inhalable	ClinicalTrials.gov, (2025b)
	2013	Phase I	*Staphylococcus aureus, Escherichia coli and Pseudomonas aeruginosa*	42	P.C.	4 mL (10^9^ PFU/mL)	Ultrasonic debridement device	Rhoads et al. (2009)
Canada	2024	Phase I/II	*Staphylococcus aureus*	1	P.C.	N.E.	Intra-articular and intravenous	ClinicalTrials.gov, (2023a)
	2023	Phase I/II	*Escherichia coli*	N.E	P.C.	N.E.	Oral, bladder, and topical	ClinicalTrials.gov, (203b)
Spain	2024	N.E.	*Staphylococcus aureus and Pseudomonas aeruginosa*	2	M.S.	N.E.	Inhalable	Bernabéu-Gimeno et al. (2024)
Asia	2016	N.E.	*Escherichia coli*	120	P.C.	1.4 × 10^9^ and 3.6 × 10^8^ PFU	Oral	Sarker et al. (2016)
Switzerland	2005	N.E.	*Escherichia coli*	15	M.S.	10^3^–10^5^ PFU/mL	Oral	Bruttin and Brüssow (2005)

N.E., Non specificized., “n”. Number of patients in the study, P.C., Phage cocktail., M.S., monophages solution.

Although the number of clinical trials has increased over the last 20 years, researchers agree that a standardized foundation for such trials needs to be established. Bacteriophages should be thoroughly tested to ensure they are properly identified and have demonstrated efficacy both *in vitro* and *in vivo* studies. Additionally, it is essential to ensure that bacteriophages can reach the host cell, quantify the number of phages arriving at the target site, and understand their pharmacological characteristics. Some authors suggest the need for a more up-to-date quantification methods that can automatically identify and count the number of phages in a sample, as traditional methods, such as quantifying lytic plaques, may lead to errors. There is also concern about the interaction between bacteriophages and antibiotics, specifically the ability of bacteria to mutate or become resistant to bacteriophages. Furthermore, some researchers are focused on the immune system’s response ton PT. Although several researchers have published on these topics, future clinical trials should aim to address them comprehensibly, depending on the specific virus they are investigating (Hitchcock et al., 2023; Nang et al., 2023).

## 11 Conclusion

Microbial resistance is one of the most critical global health challenges, with the WHO projecting it to become the most significant medical issue by 2030. To address this growing threat, various solutions must be developed to mitigate its impact. In this context, phage therapy has emerged as a promising alternative, demonstrating efficacy in certain cases and gaining traction in several countries, particularly in Europe. Nevertheless, intensive research is still needed to enhance its safety and efficacy, improve pharmaceutical processes (such as achieving higher bacteriophage titers and efficient downstream methodologies to obtain highly purified and stable products), establish regulatory frameworks, and foster social acceptance. These steps are essential to promote the widespread adoption of phage therapy globally.
